# Supplementary material for: Origin and functional diversification of PAS domain, a ubiquitous intracellular sensor
Source: Sci Adv. 2023 Aug 30;9(35):eadi4517. doi: 10.1126/sciadv.adi4517 (PMC10468136; doi:10.1126/sciadv.adi4517)
Supplement: Supplementary file 1 — Figs. S1 to S10 Tables S1 to S5 Legends for data S1 to S8 References [file sciadv.adi4517_sm.pdf]

Science Advances
AAAS

Supplementary Materials for

Origin and functional diversification of PAS domain, a ubiquitous intracellular sensor

Jiawei Xing *et al.*

Corresponding author: Igor B. Zhulin, jouline.1@osu.edu

Sci. Adv. **9**, eadi4517 (2023)
DOI: 10.1126/sciadv.adi4517

The PDF file includes:

Figs. S1 to S10
Tables S1 to S5
Legends for data S1 to S8
References

Other Supplementary Material for this manuscript includes the following:

Data S1 to S8

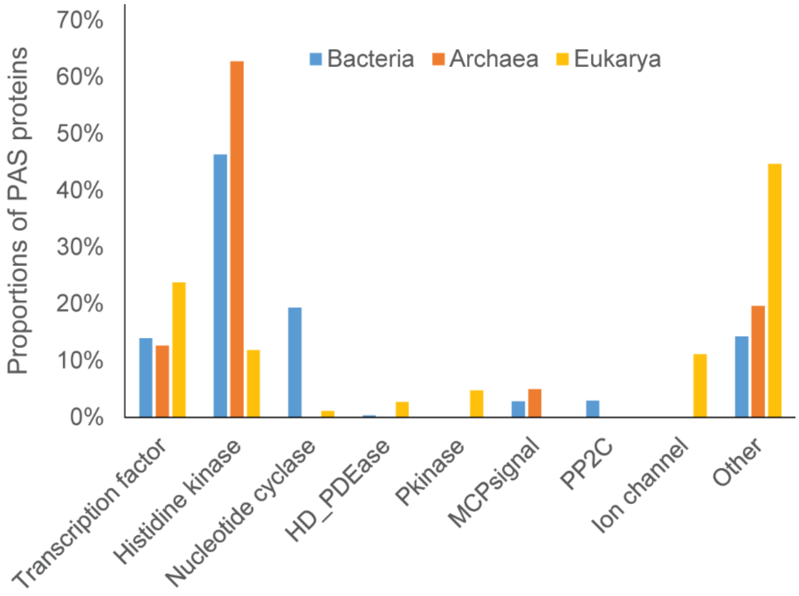

Fig. S1. Different PAS containing proteins across bacteria, archaea, and eukaryotes. Categories are defined by Pfam families. Transcription factor: HTH, HLH, GerE, GATA, Zn_clus, and Trans_reg_C; Histidine kinase: HisKA, HATPase_c, HWE, and His_kinase; Nuleotide cyclase: GGDEF, EAL, and Guanylate_cyc; HD_PDEase: HD and PDEase; Pkinase: Pkinase and PK_Tyr_Ser-Thr; MCPsignal: MCPsignal; PP2C: SpoIIE and PP2C; Ion channel: ion_trans.

A

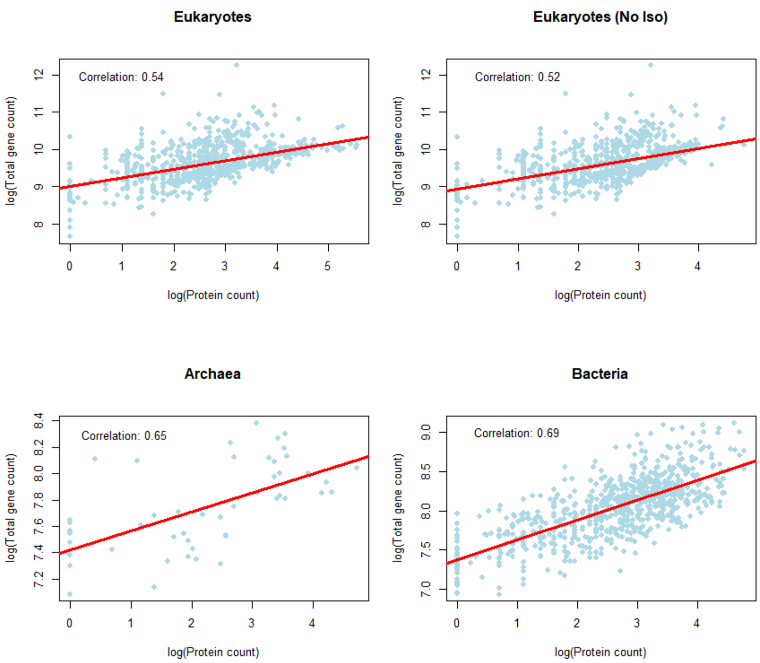

B

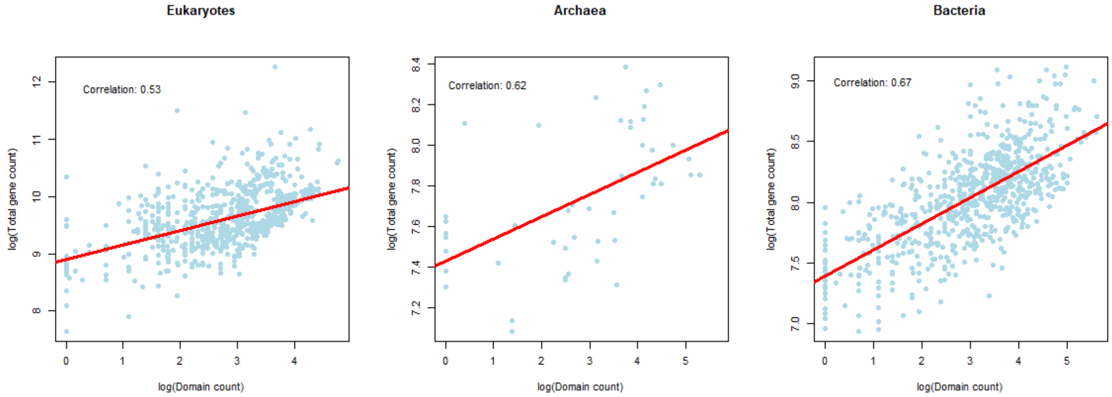

Fig. S2. Spearman correlation of PAS proteins (A) and PAS domains (B) with the total number of genes. Counts were normalized by total gene numbers at the family level (see Materials and Methods). Double-logarithmic scale is used. "No iso" indicates protein counts after removing isoforms. **A**. Eukaryotes: rho = 0.54, p-value < 2.2e-16, Eukaryotes (no iso): rho = 0.52, p-value < 2.2e-16, Archaea: rho = 0.65, p-value = 7.333e-07, Bacteria: rho = 0.69, p-value < 2.2e-16. **B**. Eukaryotes: rho = 0.53, p-value < 2.2e-16, Archaea: rho = 0.62, p-value = 2.871e-06, Bacteria: rho = 0.67, p-value < 2.2e-16. In the case of eukaryotes, the longest isoforms were used to illustrate the correlation of PAS domains with the total number of genes.

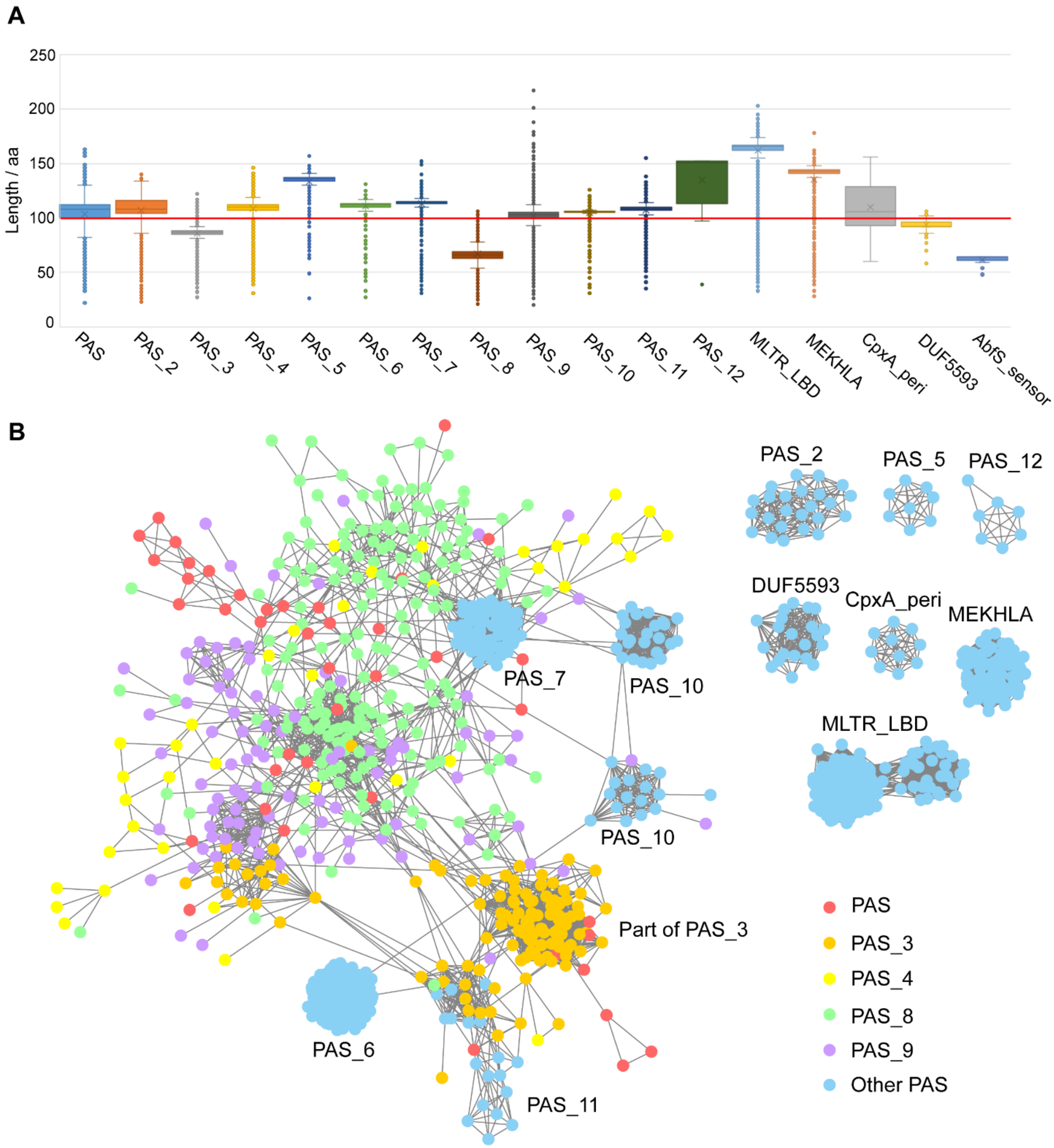

Fig. S3. PAS domain families in the Pfam database. (**A**) **Sequence lengths of PAS families.** The length of 100 amino acids is labeled by a red line. Sequences from PAS_3, PAS_8, and AbfS_sensor are substantially shorter than normal PAS domains. (**B**) **Sequence similarity network of seed sequences.** Mutual BLAST hits (E-value < 0.05, query coverage > 80%) are shown in Cytoscape. Blue nodes show separated families; other colors show overlapped families. Outlier sequences are not shown for simplicity.

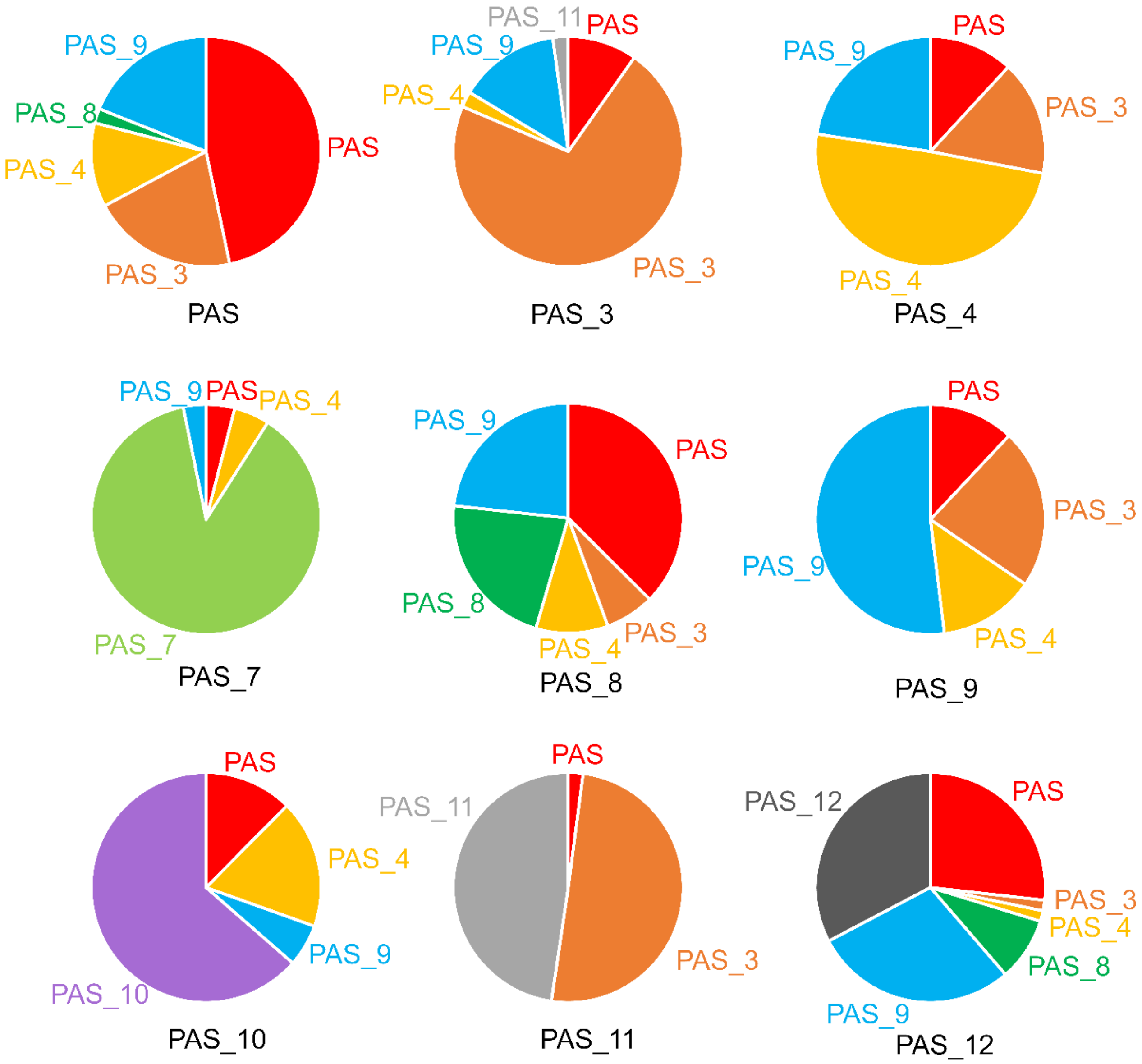

Fig. S4. Overlaps among Pfam PAS domain families. PAS domain sequences collected from RefSeq were searched against all Pfam families using *hmmscan*. Charts show the best matched HMM for sequences from each PAS family. The query HMM used for sequence collection are labeled below each chart. The rest of PAS families have few overlaps and are therefore not shown.

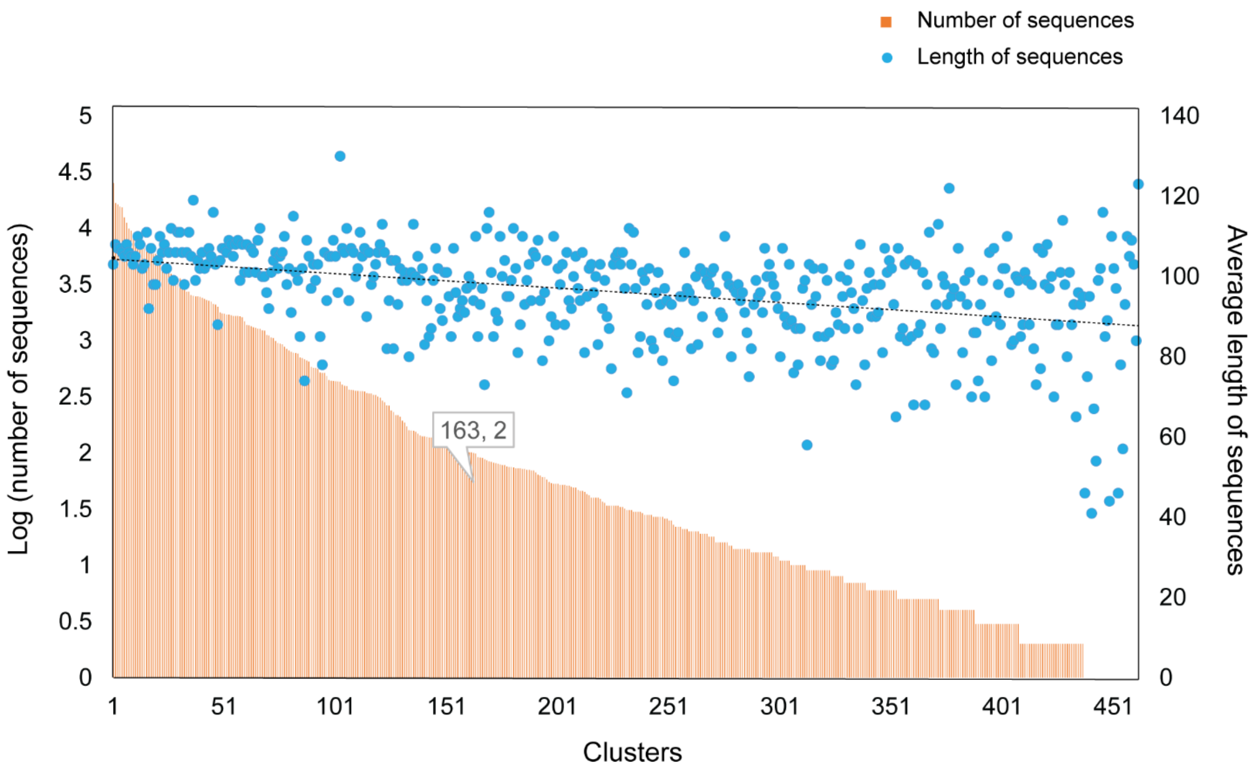

Fig. S5. Size and sequence length of PAS domain clusters. Clusters 1-462 were ordered by their numbers of PAS domain sequences from large to small. Yellow bars show the number of sequences in each cluster in the 10 based logarithm. Blue dots show the average length of sequences in each cluster. Clusters 1-163 contain at least 100 sequences, which is labeled in the plot.

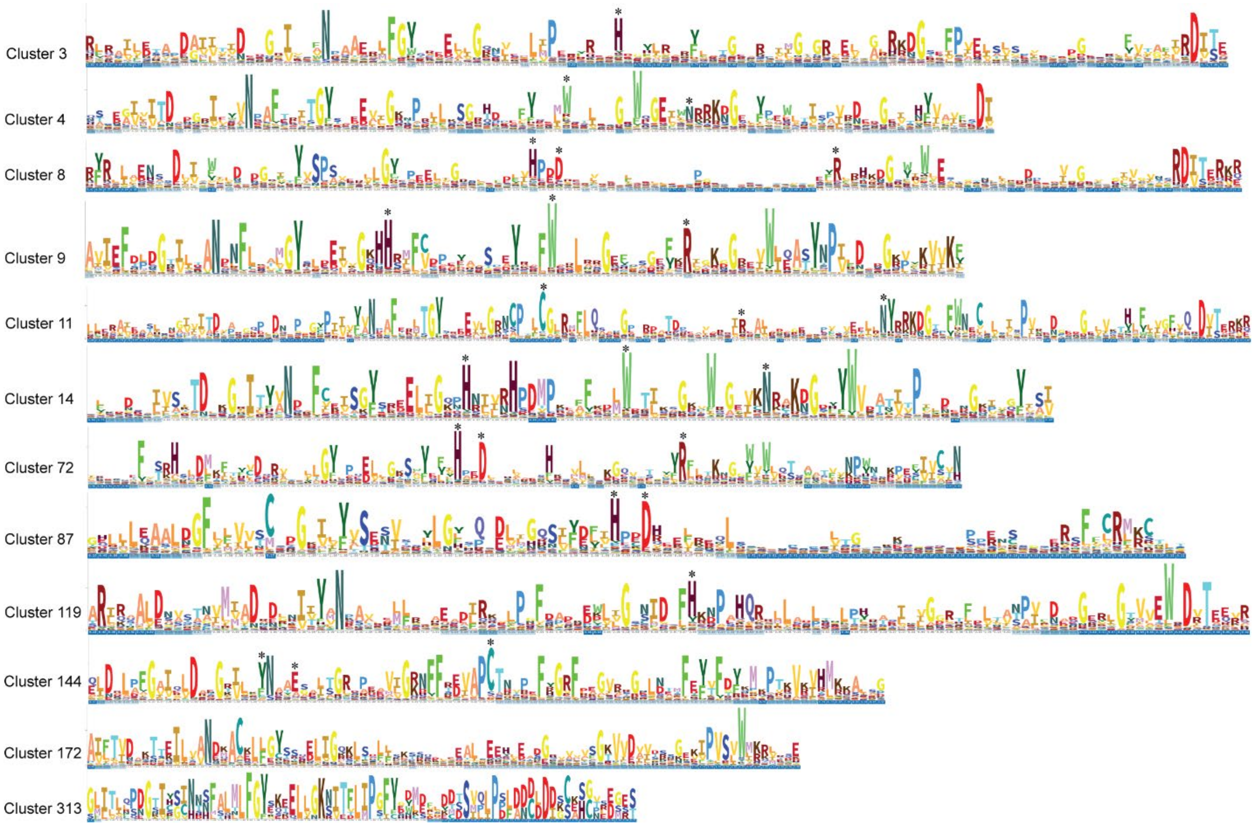

Fig. S6. Sequence logos of selected clusters. Gaps were trimmed by trimAl. Logos were built by Skylign. Conserved key residues are labeled with asterisks.

A

```
Cluster 3:
                                                            .        *
FixL-1drm RETHLRSILHTIPDAMIVIDGHGIIQLFSTAAERLFGWSELEAIGQNVNILMPEPDRSRH
FixL-1lsw RETHLRSILHTIPDAMIVIDGHGIIQLFSTAAERLFGWSELEAIGQNVNILMPEPDRSRH
FixL-1dp6 RETHLRSILHTIPDAMIVIDGHGIIQLFSTAAERLFGWSELEAIGQNVNILMPEPDRSRH
FixL-1d06 RDAHLRSILDTVPDATVVSATDGTIVSFNAAAVRQFGYAEEEVIGQNLRILMPEPYRHEH
FixL-1ew0 RDAHLRSILDTVPDATVVSATDGTIVSFNAAAVRQFGYAEEEVIGQNLRILMPEPYRHEH
DosP-1v9z ----------------VLINENDEVMFFNPAAEKLWGYKREEVIGNNIDMLIPRDLRPAH
DosP-1s66 ----------------VLINENDEVMFFNPAAEKLWGYKREEVIGNNIDMLIPRDLRPAH

            .                .
FixL-1drm DSYISRYRTTSDPHIIGIGRIVTGKRRDGTTFPMHLSIGEMQSGGEPYFTGFVRDLTEH-
FixL-1lsw DSYISRYRTTSDPHIIGIGRIVTGKRRDGTTFPMHLSIGEMQSGGEPYFTGFVRDLTEH-
FixL-1dp6 DSYISRYRTTSDPHIIGIGRIVTGKRRDGTTFPMHLSIGEMQSGGEPYFTGFVRDLTEH-
FixL-1d06 DGYLQRYMATGEKRIIGIDRVVSQQRKDGSTFPMKLAVGEMRSGGERFFTGFIRDLTER-
FixL-1ew0 DGYLQRYMATGEKRIIGIDRVVSQQRKDGSTFPMKLAVGEMRSGGERFFTGFIRDLTER-
DosP-1v9z PEYIRHNREGGKARVEGMSRELQLEKKDGSKIWTRFALSKVSAEGKVYYLALVRDASVEM
DosP-1s66 PEYIRHNREGGKARVEGMSRELQLEKKDGSKIWTRFALSKVSAEGKVYYLALVRDASVEM

Cluster 119:
           . .                              .           .  .*.   ..
Aer2-4hi4 VMIADNDLNIIYMNRTVSEMLGRAEADIRKQLPNFDAGR--LMGANIDVFHKNPAHQRHL
Aer2-3vol VMIADNDLNIIYMNRTVSEMLGRAEADIRKQLPNFDAGR--LMGANIDVFHKNPAHQRHL
Aer2-6ceq LMMADKEGIIQYLNPALLQLLTHREPELAQAFPGFKAAE--LVGKNIDIFHKNPAHQRSI

                                                  .
Aer2-4hi4 LANLTGV-HKAELNLGGRRFSLDVVPVFNDANERLGSAVQWTDRTE
Aer2-3vol LANLTGV-HKAELNLGGRRFSLDVVPVFNDANERLGSAVQWTDRTE
Aer2-6ceq ISNPERLPFTSMIKVGSLEFNLTCIAMRDTKGEYIGPALQWVDITE
```

B

```
Clusters 4(NifL), 14(Aer, MmoS), 9(AerC), and 11(Phot1):
                                                        .
NifL-2gj3  EIFRQTVEHAPIAISITDLKANILYANRAFRTITGYGSEEVLGKNES-ILSNGTTPRLVY
Aer-8dik   ------------LMSTTDLQSYITHANDTFVQVSGYTLQELQGQPHN-MVRHPDMPKAAF
MmoS-3ewk  -------------VSITDLQGRILYANDNFCAVSRYGREELVGQDHR-IVNSGYHGKAYI
AerC-PAS A ----------SNGIITFDTYGNILSANDQFLRCMGYNLEEIKGKHHRIFVDPKLHGSLDY
AerC-PAS B ------------AVIEFTPDGTVITANENFLSLLGYTLREIEGRHHSTFVDPAEHGGADY
Phot1-2z6c ----------FVVSDATKPDYPIMYASAGFFNMTGYTSKEVVGRNCR-FLQGSGTDADEL

              *              .
NifL-2gj3  QALWGRLAQKKPWSGVLVNRRKDKTLYLAELTVAPVLNEAGETIYYLGMHRD-------
Aer-8dik   ADMWFTLKKGEPWSGIVKNRRKNGDHYWVRANAVPMV-REGKISGYMSIRTRATDEEIA
MmoS-3ewk  RDMWRTISRGNIWQGEFCNRRKDGTRYWVDSTIVPLMDNAGKPRQYISIRRDITA----
AerC-PAS A EDFWERLRRGEFQSSLYKRIGKGGREVWIEASYNPIKNRQGVTHKVVKVCTDVTE----
AerC-PAS B RAFWESLRQGRFQAAQYKRIGKGGRVVWIQASYNPVFDTSNRLSKIVKFATDIT-----
Phot1-2z6c AKIRETLAAGNNYCGRILNYKKDGTSFWNLLTIAPIKDESGKVLKFIGMQVEVSK----
```

Fig. S7. Cofactor-binding residues from PDB structures. (**A**) Multiple sequence alignment of heme-binding PAS domains from clusters 3 and 119. (**B**) Multiple sequence alignment of flavin-binding PAS domains from clusters 4, 14, 9, and 11. Residues within 4.0 Å of cofactors are highlighted in gray; residues directly interacting with cofactors by hydrogen bonds or hydrophobic interactions are highlighted in yellow. Conserved residues directly interacting with cofactors in all structures are labeled by asterisks; other conserved residues located nearby cofactors are labeled by dots.

A

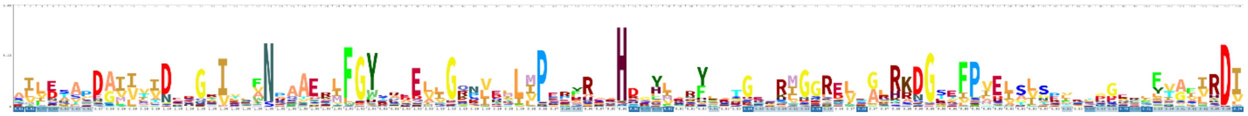

B

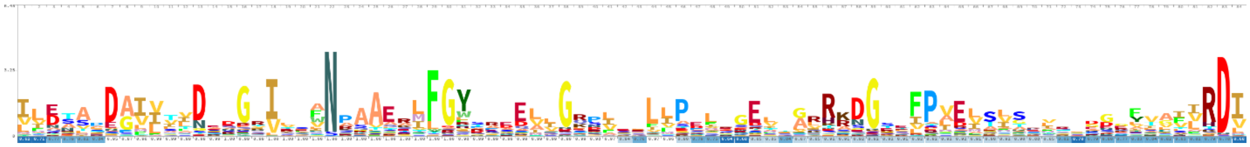

Fig. S8. Sequence conservation of cluster 3. (**A**) Sequences with conserved His for heme binding. (**B**) Sequences without His for heme binding. Logos are made by Skylign.

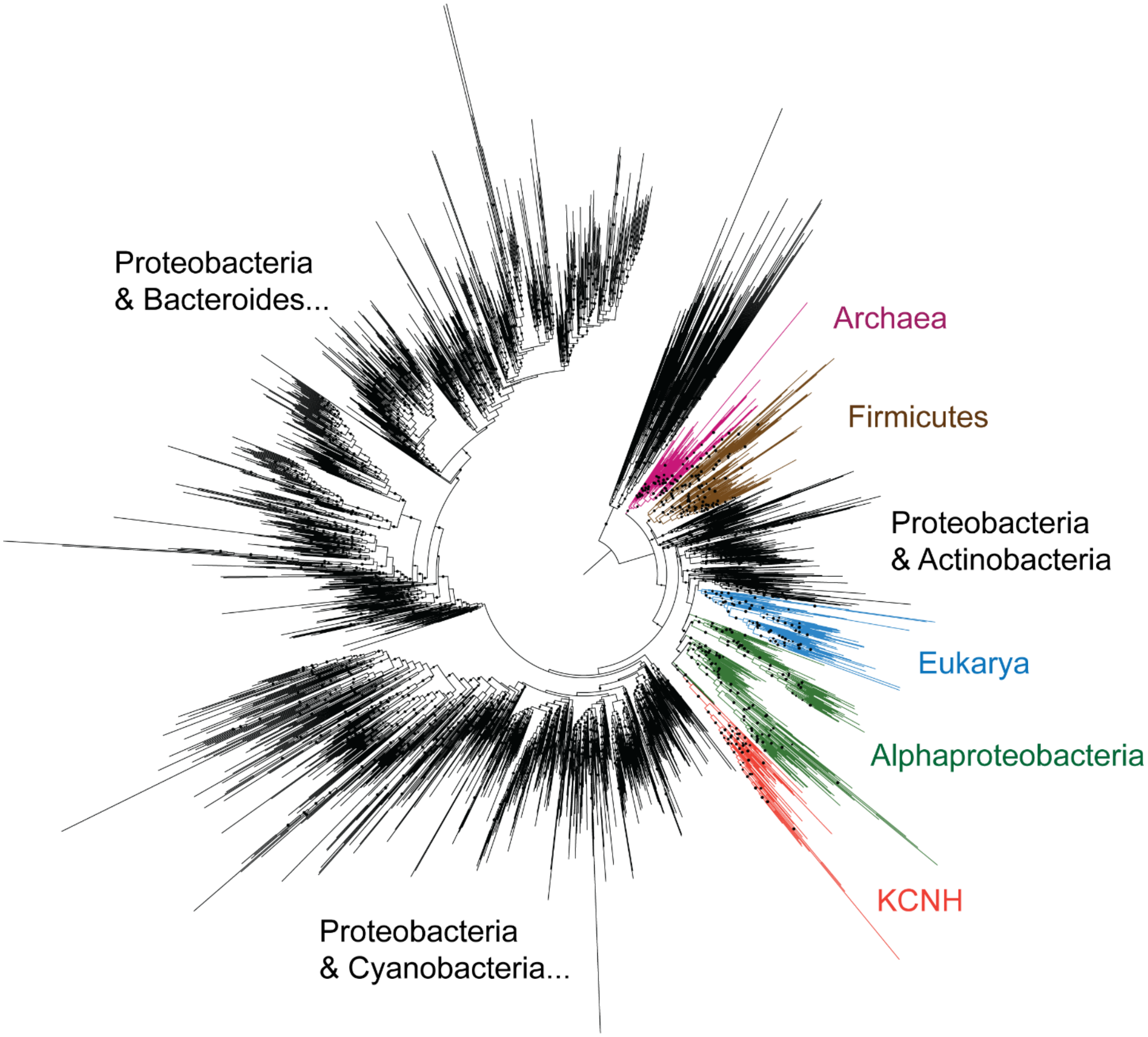

Fig. S9. Phylogenetic tree of cluster 11 (FMN). Sequences from cluster 11 were reduced at 60% redundancy. The maximum likelihood tree was built using LG+G4 model. Dots show bootstrap values > 70. Taxonomy was highlighted and labeled beside the tree. KCNH-PAS is close to PAS from Alphaproteobacteria.

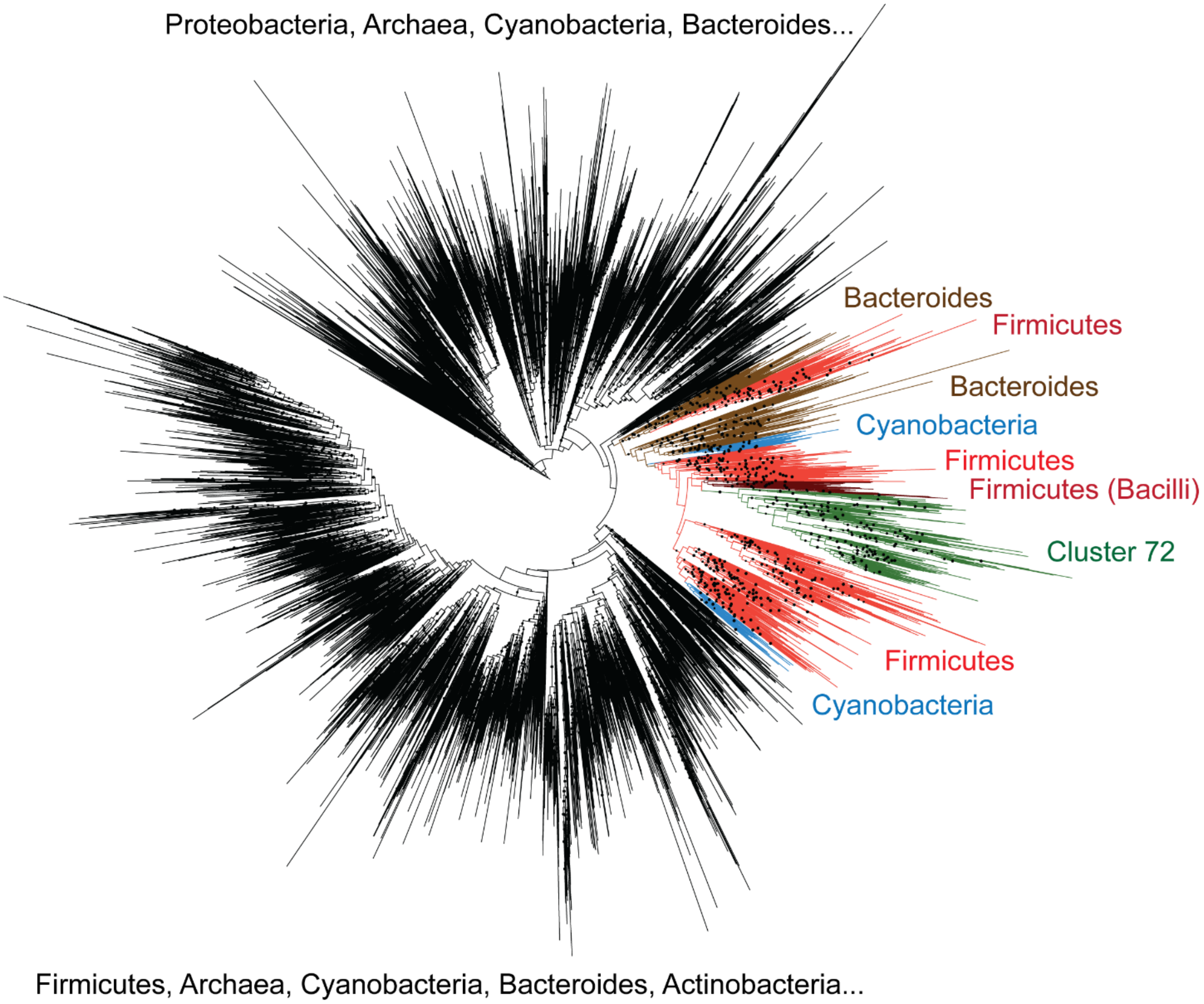

Fig. S10. Phylogenetic tree of clusters 8 and 72 (bHLH-PAS B). Sequences from cluster 8 and 72 were reduced at 60% redundancy. The maximum likelihood tree was built using LG+G4 model. Dots show bootstrap values > 70. Taxonomy was highlighted and labeled beside the tree. Cluster 72 is close to PAS from Firmicutes (Bacilli).

Table S1 Cofactor-binding PAS domains

Cofactors	Proteins	Example organisms	Pfam	Clusters	PDB	References
Heme	FixL	*Bradyrhizobium japonicum*	PAS	3	1DRM	(*27*)
	DosP	*Escherichia coli*	PAS_9	3	1V9Y	(*28*)
	Aer2	*Pseudomonas aeruginosa*	PAS_8	119	4HI4	(*29, 79*)
	CLOCK	*Homo sapiens*	PAS		-	(*42*)
	NPAS2	*Homo sapiens*	PAS		-	(*43*)
	ERG3	*Homo sapiens*	PAS_9		-	(*41*)
	RcoM	*Burkholderia xenovorans*	PAS_4?		-	(*80*)
	PDEA1	*Acetobacter xylinum*	PAS_9		-	(*81*)
	YybT	*Bacillus subtilis*	-		-	(*82*)
	NtrY	*Brucella abortus*	PAS		-	(*83*)
	Hpk2	*Treponema denticola*	PAS_4		-	(*84*)
	SenX3	*Mycobacterium tuberculosis*	PAS_4?		-	(*85*)
	PpsR	*Rhodobacter sphaeroides*	PAS_9		-	(*86*)
	BdlA	*Pseudomonas aeruginosa*	PAS_9		-	(*87*)
	IcpB	*Azorhizobium caulinodans*	PAS_9		-	(*88*)
	Per2	*Mus musculus*	PAS, PAS_3		-	(*44, 89, 90*)
FAD	Aer	*Escherichia coli*	PAS_3	14	8DIK	(*32, 91*)
	CetB	*Campylobacter jejuni*	PAS_3	14	-	(*92*)
	MmoS	*Methylococcus capsulatus*	PAS_9	14	3EWK	(*31*)
	NifL	*Azotobacter vinelandii*	PAS	4	2GJ3	(*30*)
	Vivid	*Neurospora crassa*	PAS_9	11	2PDR	(*93*)
	WC-1	*Neurospora crassa*	PAS_9	11	-	(*9, 94*)
	AerC	*Azospirillum brasilense*	PAS_9, PAS_3	9	-	(*33*)
FMN	YtvA	*Bacillus subtilis*	PAS_9	11	2PR6	(*95*)
	phototropins	*Arabidopsis thaliana*	PAS_9	11	2Z6C	(*96*)
	phy3	*Adiantum capillus-veneris*	PAS_9	11	1G28	(*97*)
	LOV	*Rhodobacter sphaeroides*	PAS_9	11	4HIA	(*98*)
	PpSB-LOV	*Pseudomonas putida*	PAS_9	11	5J4E	(*99*)
	DsLOV	*Dinoroseobacter shibae*	PAS_9	11	4KUO	(*100*)
	LovK	*Caulobacter cresentus*	PAS_9	11	-	(*101*)
	Lovhk	*Brucella abortus*	PAS_9	11	5EPV	(*102*)
	11EL222	11*Erythrobacter litoralis*	PAS_9	11	3P7N	(*103*)
	Cagg_3753	*Chloroflexus aggregans*	PAS_3	11	6RHG	(*104*)
	aureochrome	*Phaeodactylum tricornutum*	PAS_9	11	5DKK	(*105*)
	PAL	*Nakamurella multipartita*	PAS_9	11	6HMJ	(*106*)
	ENV1	*Trichoderma reesei*	PAS_9	11	4WUJ	(*107*)
p-coumaric acid	PYP	*Halorhodospira halophila*	PAS	144	2PHY	(*37*)
	Ppr	*Rhodospirillum centenum*	PAS?	144	1MZU	(*108*)
	Ppd	*Thermochromatium tepidum*	PAS	144	-	(*109*)
Riboflavin	EL346	*Erythrobacter litoralis*	PAS_9	11	4R38	(*36*)
Fe-S cluster	NreB	*Staphylococcus carnosus*	-	3	-	(*53*)

Questions marks indicate PAS domains below the Pfam cutoff threshold. Heme binding in some PAS domains are inconclusive and their clusters were not shown.

Table S2. Distribution of Pfam PAS families in Bacteria, Archaea, and Eukaryotes

PAS family	Bacteria	Archaea	Eukaryotes
PAS	Yes	Yes	Yes
PAS_2	Yes	No	Yes
PAS_3	Yes	Yes	Yes
PAS_4	Yes	Yes	Yes
PAS_5	Yes	No	Yes
PAS_6	Yes	No	Yes
PAS_7	Yes	Yes	No
PAS_8	Yes	Yes	Yes
PAS_9	Yes	Yes	Yes
PAS_10	Yes	Yes	No
PAS_11	Yes	No	Yes
PAS_12	Yes	No	No
MLTR_LBD	Yes	No	No
MEKHLA	Yes	No	Yes
DUF5593	Yes	No	No
CpxA_peri	Yes	No	No
AbfS_sensor	Yes	No	No

Table S3. PAS secondary structure elements in Pfam PAS HMMs.

PAS family	Secondary structures															
PAS			A’	A	B		C	D	E	F			G	H	I	
PAS_2			A’	A	B		C	D	E	F			G	H	I	
PAS_3				*	B		C	D	E	F			G	H	I	
PAS_4				A	B		C	D	E	F			G	H	I	J
PAS_5	A’’’	A’’	A’	A	B		C		E	F			G	H	I	
PAS_6			A’	A	B					F			G	H	I	J
PAS_7				A	B		C	D	E	F			G	H	I	J
PAS_8			A’	A	B		C	D	E	F			*	*	*	
PAS_9				A	B		C	D	E	F			G	H	I	
PAS_10			A’	A	B		C	D	E	F			G	H	I	
PAS_11				A	B		C	D	E	F			G	H	I	J
PAS_12	A’’’	A’’	A’	A	B		C	D	E	F			G	H	I	
MLTR_LBD			A’	A	B		C	D	E	F	F’	F’’	G	H	I	J
MEKHLA		A’’	A’	A			C	D	E	F			G	H	I	
DUF5593				A	B	C			E	F			G	H	I	
CpxA_peri**		A’’	A’	A	B					F			G	H	I	J
AbfS_sensor**		A’’	A’	A			C	D	E				*	*	*	

PAS domains have a common structural fold: Aß-Bß-Cα-Dα-Eα-Fα-Gß-Hß-Iß. The representative sequence of each Pfam PAS family was identified using *hmmsearch* against seed sequences. Secondary structures for representative sequences were identified using Quick2D (https://toolkit.tuebingen.mpg.de/tools/quick2d).
*Essential regions in PAS_3, PAS_8, and AbfS_sensor were not covered by Pfam HMMs.
**Two extracellular Cache domains were mistakenly included in the PAS superfamily.

Table S4. Overlaps among Pfam PAS domain families

PAS family	RefSeq sequences	Correct sequences	Percentage of correctness
PAS*	648,279	300,617	46%
PAS_2	11,734	11,734	100%
PAS_3*	481,799	344,493	72%
PAS_4*	641,254	313,073	49%
PAS_5	4,049	4,049	100%
PAS_6	11,407	11,402	100%
PAS_7*	64,296	55,951	87%
PAS_8*	241,387	52,895	22%
PAS_9*	577,171	299,371	52%
PAS_10*	23,131	14,674	63%
PAS_11*	35,694	16,956	48%
PAS_12*	269	88	33%
MLTR_LBD	58,261	58,200	100%
MEKHLA	3,572	3,262	91%
DUF5593	1, 524	1,524	100%
CpxA_peri	2,575	2,575	100%
AbfS_sensor	93	93	100%

Sequences for each PAS family were collected from RefSeq by *hmmsearch* using Pfam HMMs as queries (E-value < 0.01). For some PAS families, query HMMs also matched sequences from other families. Correct sequences are defined as sequences with the best match to the query HMM using *hmmscan* (Fig. S4).
*Nine PAS families have overlapped sequences.

Table S5 PAS proteins in the human proteome.

Class	Protein	Function	Associated Disease	Ref
KCNH	ERG1 (KCNH2)	Heart rhythm	Long/short QT syndrome	(*110, 111*)
	ERG2 (KCNH6)	Expressed in CNS		(*112*)
	ERG3 (KCNH7)			(*112*)
	EAG1 (KCNH1)		Cancer, Temple-Barrister syndrome, Zimmerman-Laband syndrome	(*113-115*)
	EAG2 (KCNH5)		Cancer	(*113, 116*)
	ELK1 (KCNH4)			(*117*)
	ELK2 (KCNH3)			(*117*)
	ELK3 (KCNH8)			(*118*)
PDE8	PDE8A	cAMP hydrolyzation	Depression	(*59, 119*)
	PDE8B		Adrenal hyperplasia	(*120*)
bHLH-PAS	AHR	Ligand sensing	Cancer, immune diseases	(*13, 121, 122*)
	AHRR	AHR repressor	Cancer	(*123*)
	HIF1α	Hypoxia responses	Cancer, cardiovascular diseases	(*10, 12, 124*)
	HIF2α (EPAS1)		Cancer, erythrocytosis	(*14, 125, 126*)
	HIF3α (IPAS)	HIF repressor	Cancer, Parkinson's disease	(*127-129*)
	SIM1	Neural development	Obesity	(*130*)
	SIM2		Cancer, Down syndrome	(*131-133*)
	NPAS1		Psychiatric diseases	(*134, 135*)
	NPAS3		Schizophrenia	(*136, 137*)
	NPAS4	Neural development, DNA repair	Schizophrenia, diabetes, aging	(*138-140*)
	CLOCK	Circadian rhythm	Circadian disorders	(*141*)
	NPAS2		Circadian disorders	(*142*)
	PASD1	CLOCK repressor, cancer testis antigen	Circadian disorders, multiple myeloma, lymphoma	(*143-145*)
	ARNT	bHLH dimerization	Cancer	(*146*)
	ARNT2		Cancer	(*146*)
	BMAL1 (ARNTL)		Circadian disorders	(*147*)
	BMAL2 (ARNTL2)		Circadian disorders	(*148*)

	NCOA1 (SRC1)	Transcriptional coactivator	Cancer	(*149, 150*)
	NCOA2 (SRC2)		Metabolic diseases	(*151*)
	NCOA3 (SRC3)		Cancer, hearing loss	(*152, 153*)
	PER1*	Circadian rhythm	Circadian disorders, long-term memory loss, hypertension	(*154, 155*)
	PER2*		Circadian disorders, cardiovascular diseases	(*156*)
	PER3*		Circadian disorders, mood disorders	(*157*)
PASK	PASK	Signal transduction	Metabolic diseases	(*158*)

* PER1-3 do not have bHLH domains but function together with bHLH-PAS proteins.

Dataset S1. Presence of PAS domains in UniProt reference proteomes.

Data contains 22,925 reference proteomes from UniProt (*65*). PAS domain presence in each proteome is shown.

Dataset S2. PAS domain containing proteins identified in InterPro.

Data contains all PAS-containing proteins from InterPro defined by the Pfam PAS fold (*18, 23*).

Dataset S3. PAS domains identified in 14 representative eukaryotic genomes.

Data contains a summary sheet and 14 sheets with PAS-containing proteins in each genome.

Clusters to which PAS domains belong are shown in the file. PAS domains that belong to well-defined clusters and have conserved key residues are highlighted (blue, heme-binding; red, flavin-binding). Isoforms are highlighted in red.

Dataset S4. PAS domains in 43 representative eukaryotic genomes.

Data contains a summary sheet and 43 sheets with PAS-containing proteins in each genome. Isoforms and false positive hits are highlighted in red.

Dataset S5. RefSeq accession numbers for proteins with PAS domains assigned to MCL clusters.

Each column contains PAS domains of a cluster.

Dataset S6. Phyletic distribution and domain architecture of PAS domain containing proteins in the RefSeq database.

Each sheet contains PAS-containing proteins from a well-defined cluster with the conserved key residues for cofactor-binding.

Dataset S7. PAS domains in the human genome identified by the structure search.

Data contains human proteins matching to 2KDK with TMscore larger than 0.5 (*78*).

Dataset S8. Results of BLAST searches with representative eukaryotic PAS domains.

Each sheet contains the top 100 BLAST hits using a eukaryotic PAS domain as query (results are ordered by sequence identities). The top BLAST hits from bacterial proteins are highlighted in yellow.

REFERENCES AND NOTES

1. B. L. Taylor, I. B. Zhulin, PAS domains: Internal sensors of oxygen, redox potential, and light. *Microbiol. Mol. Biol. Rev.* **63**, 479–506 (1999).

2. A. A. Upadhyay, A. D. Fleetwood, O. Adebali, R. D. Finn, I. B. Zhulin, Cache domains that are homologous to, but different from PAS domains comprise the largest superfamily of extracellular sensors in prokaryotes. *PLOS Comput. Biol.* **12**, e1004862 (2016).

3. M. B. Neiditch, M. J. Federle, S. T. Miller, B. L. Bassler, F. M. Hughson, Regulation of LuxPQ receptor activity by the quorum-sensing signal autoinducer-2. *Mol. Cell* **18**, 507–518 (2005).

4. J. T. Henry, S. Crosson, Ligand-binding PAS domains in a genomic, cellular, and structural context. *Annu. Rev. Microbiol.* **65**, 261–286 (2011).

5. B. L. Taylor, I. B. Zhulin, M. S. Johnson, Aerotaxis and other energy-sensing behavior in bacteria. *Annu. Rev. Microbiol.* **53**, 103–128 (1999).

6. J. E. Silpe, B. L. Bassler, A host-produced quorum-sensing autoinducer controls a phage lysis-lysogeny decision. *Cell* **176**, 268–280.e13 (2019).

7. I. R. Monk, N. Shaikh, S. L. Begg, M. Gajdiss, L. K. R. Sharkey, J. Y. H. Lee, S. J. Pidot, T. Seemann, M. Kuiper, B. Winnen, R. Hvorup, B. M. Collins, G. Bierbaum, S. R. Udagedara, J. R. Morey, N. Pulyani, B. P. Howden, M. J. Maher, C. A. McDevitt, G. F. King, T. P. Stinear, Zinc-binding to the cytoplasmic PAS domain regulates the essential WalK histidine kinase of Staphylococcus aureus. *Nat. Commun.* **10**, 3067 (2019).

8. S. M. Harper, L. C. Neil, K. H. Gardner, Structural basis of a phototropin light switch. *Science* **301**, 1541–1544 (2003).

9. A. C. Froehlich, Y. Liu, J. J. Loros, J. C. Dunlap, White Collar-1, a circadian blue light photoreceptor, binding to the frequency promoter. *Science* **297**, 815–819 (2002).

10. G. L. Semenza, Hypoxia-inducible factors in physiology and medicine. *Cell* **148**, 399–408 (2012).

11. B. Stockinger, P. Di Meglio, M. Gialitakis, J. H. Duarte, The aryl hydrocarbon receptor: Multitasking in the immune system. *Annu. Rev. Immunol.* **32**, 403–432 (2014).

12. G. L. Semenza, Targeting HIF-1 for cancer therapy. *Nat. Rev. Cancer* **3**, 721–732 (2003).

13. I. A. Murray, A. D. Patterson, G. H. Perdew, Aryl hydrocarbon receptor ligands in cancer: Friend and foe. *Nat. Rev. Cancer* **14**, 801–814 (2014).

14. E. Jonasch, F. Donskov, O. Iliopoulos, W. K. Rathmell, V. K. Narayan, B. L. Maughan, S. Oudard, T. Else, J. K. Maranchie, S. J. Welsh, S. Thamake, E. K. Park, R. F. Perini, W. M. Linehan, R. Srinivasan; MK-6482-004 Investigators, Belzutifan for renal cell carcinoma in von hippel-lindau disease. *N. Engl. J. Med.* **385**, 2036–2046 (2021).

15. V. M. Gumerov, E. P. Andrianova, M. A. Matilla, K. M. Page, E. Monteagudo-Cascales, A. C. Dolphin, T. Krell, I. B. Zhulin, Amino acid sensor conserved from bacteria to humans. *Proc. Natl. Acad. Sci. U.S.A.* **119**, e2110415119 (2022).

16. I. B. Zhulin, B. L. Taylor, R. Dixon, PAS domain S-boxes in Archaea, Bacteria and sensors for oxygen and redox. *Trends Biochem. Sci.* **22**, 331–333 (1997).

17. C. P. Ponting, L. Aravind, PAS: A multifunctional domain family comes to light. *Curr. Biol.* **7**, R674–R677 (1997).

18. T. Paysan-Lafosse, M. Blum, S. Chuguransky, T. Grego, B. L. Pinto, G. A. Salazar, M. L. Bileschi, P. Bork, A. Bridge, L. Colwell, J. Gough, D. H. Haft, I. Letunić, A. Marchler-Bauer, H. Mi, D. A. Natale, C. A. Orengo, A. P. Pandurangan, C. Rivoire, C. J. A. Sigrist, I. Sillitoe, N. Thanki, P. D. Thomas, S. C. E. Tosatto, C. H. Wu, A. Bateman, InterPro in 2022. *Nucleic Acids Res.* **51**, D418–D427 (2023).

19. S. R. Eddy, Accelerated profile HMM searches. *PLoS Comput. Biol.* **7**, e1002195 (2011).

20. M. van Kempen, S. S. Kim, C. Tumescheit, M. Mirdita, J. Lee, C. L. M. Gilchrist, J. Söding, M. Steinegger, Fast and accurate protein structure search with Foldseek. *Nat. Biotechnol.*, (2023).

21. J. Jumper, R. Evans, A. Pritzel, T. Green, M. Figurnov, O. Ronneberger, K. Tunyasuvunakool, R. Bates, A. Žídek, A. Potapenko, A. Bridgland, C. Meyer, S. A. A. Kohl, A. J. Ballard, A. Cowie, B. Romera-Paredes, S. Nikolov, R. Jain, J. Adler, T. Back, S. Petersen, D. Reiman, E. Clancy, M. Zielinski, M. Steinegger, M. Pacholska, T. Berghammer, S. Bodenstein, D. Silver, O. Vinyals, A. W. Senior, K. Kavukcuoglu, P. Kohli, D. Hassabis, Highly accurate protein structure prediction with AlphaFold. *Nature* **596**, 583–589 (2021).

22. Z. Lin, H. Akin, R. Rao, B. Hie, Z. Zhu, W. Lu, N. Smetanin, R. Verkuil, O. Kabeli, Y. Shmueli, A. dos Santos Costa, M. Fazel-Zarandi, T. Sercu, S. Candido, A. Rives, Evolutionary-scale prediction of atomic-level protein structure with a language model. *Science* **379**, 1123–1130 (2023).

23. J. Mistry, S. Chuguransky, L. Williams, M. Qureshi, G. A. Salazar, E. L. L. Sonnhammer, S. C. E. Tosatto, L. Paladin, S. Raj, L. J. Richardson, R. D. Finn, A. Bateman, Pfam: The protein families database in 2021. *Nucleic Acids Res.* **49**, D412–D419 (2021).

24. N. A. O'Leary, M. W. Wright, J. R. Brister, S. Ciufo, D. Haddad, R. McVeigh, B. Rajput, B. Robbertse, B. Smith-White, D. Ako-Adjei, A. Astashyn, A. Badretdin, Y. Bao, O. Blinkova, V. Brover, V. Chetvernin, J. Choi, E. Cox, O. Ermolaeva, C. M. Farrell, T. Goldfarb, T. Gupta, D. Haft, E. Hatcher, W. Hlavina, V. S. Joardar, V. K. Kodali, W. Li, D. Maglott, P. Masterson, K. M. McGarvey, M. R. Murphy, K. O'Neill, S. Pujar, S. H. Rangwala, D. Rausch, L. D. Riddick, C. Schoch, A. Shkeda, S. S. Storz, H. Sun, F. Thibaud-Nissen, I. Tolstoy, R. E. Tully, A. R. Vatsan, C. Wallin, D. Webb, W. Wu, M. J. Landrum, A. Kimchi, T. Tatusova, M. DiCuccio, P. Kitts, T. D. Murphy, K. D. Pruitt, Reference sequence (RefSeq) database at NCBI: Current status, taxonomic expansion, and functional annotation. *Nucleic Acids Res.* **44**, D733–D745 (2016).

25. B. Buchfink, C. Xie, D. H. Huson, Fast and sensitive protein alignment using DIAMOND. *Nat. Methods* **12**, 59–60 (2015).

26. A. J. Enright, S. Van Dongen, C. A. Ouzounis, An efficient algorithm for large-scale detection of protein families. *Nucleic Acids Res.* **30**, 1575–1584 (2002).

27. W. Gong, B. Hao, S. S. Mansy, G. Gonzalez, M. A. Gilles-Gonzalez, M. K. Chan, Structure of a biological oxygen sensor: A new mechanism for heme-driven signal transduction. *Proc. Natl. Acad. Sci. U.S.A.* **95**, 15177–15182 (1998).

28. H. Kurokawa, D. S. Lee, M. Watanabe, I. Sagami, B. Mikami, C. S. Raman, T. Shimizu, A redox-controlled molecular switch revealed by the crystal structure of a bacterial heme PAS sensor. *J. Biol. Chem.* **279**, 20186–20193 (2004).

29. M. V. Airola, D. Huh, N. Sukomon, J. Widom, R. Sircar, P. P. Borbat, J. H. Freed, K. J. Watts, B. R. Crane, Architecture of the soluble receptor Aer2 indicates an in-line mechanism for PAS and HAMP domain signaling. *J. Mol. Biol.* **425**, 886–901 (2013).

30. J. Key, M. Hefti, E. B. Purcell, K. Moffat, Structure of the redox sensor domain of Azotobacter vinelandii NifL at atomic resolution: Signaling, dimerization, and mechanism. *Biochemistry* **46**, 3614–3623 (2007).

31. U. E. Ukaegbu, A. C. Rosenzweig, Structure of the redox sensor domain of Methylococcus capsulatus (Bath) MmoS. *Biochemistry* **48**, 2207–2215 (2009).

32. Z. A. Maschmann, T. K. Chua, S. Chandrasekaran, H. Ibanez, B. R. Crane, Redox properties and PAS domain structure of the Escherichia coli energy sensor Aer indicate a multi-state sensing mechanism. *J. Biol. Chem.* **298**, 102598 (2022).

33. Z. Xie, L. E. Ulrich, I. B. Zhulin, G. Alexandre, PAS domain containing chemoreceptor couples dynamic changes in metabolism with chemotaxis. *Proc. Natl. Acad. Sci. U.S.A.* **107**, 2235–2240 (2010).

34. J. Herrou, S. Crosson, Function, structure and mechanism of bacterial photosensory LOV proteins. *Nat. Rev. Microbiol.* **9**, 713–723 (2011).

35. S. T. Glantz, E. J. Carpenter, M. Melkonian, K. H. Gardner, E. S. Boyden, G. K. Wong, B. Y. Chow, Functional and topological diversity of LOV domain photoreceptors. *Proc. Natl. Acad. Sci. U.S.A.* **113**, E1442–E1451 (2016).

36. G. Rivera-Cancel, W. H. Ko, D. R. Tomchick, F. Correa, K. H. Gardner, Full-length structure of a monomeric histidine kinase reveals basis for sensory regulation. *Proc. Natl. Acad. Sci. U.S.A.* **111**, 17839–17844 (2014).

37. J. L. Pellequer, K. A. Wager-Smith, S. A. Kay, E. D. Getzoff, Photoactive yellow protein: A structural prototype for the three-dimensional fold of the PAS domain superfamily. *Proc. Natl. Acad. Sci. U.S.A.* **95**, 5884–5890 (1998).

38. A. Moglich, R. A. Ayers, K. Moffat, Structure and signaling mechanism of Per-ARNT-Sim domains. *Structure* **17**, 1282–1294 (2009).

39. M. A. Spence, M. D. Mortimer, A. M. Buckle, B. Q. Minh, C. J. Jackson, A comprehensive phylogenetic analysis of the serpin superfamily. *Mol. Biol. Evol.* **38**, 2915–2929 (2021).

40. A. A. Burnim, M. A. Spence, D. Xu, C. J. Jackson, N. Ando, Comprehensive phylogenetic analysis of the ribonucleotide reductase family reveals an ancestral clade. *eLife* **11**, e79790 (2022).

41. M. J. Burton, J. Cresser-Brown, M. Thomas, N. Portolano, J. Basran, S. L. Freeman, H. Kwon, A. R. Bottrill, M. J. Llansola-Portoles, A. A. Pascal, R. Jukes-Jones, T. Chernova, R. Schmid, N. W. Davies, N. M. Storey, P. Dorlet, P. C. E. Moody, J. S. Mitcheson, E. L. Raven, Discovery of a heme-binding domain in a neuronal voltage-gated potassium channel. *J. Biol. Chem.* **295**, 13277–13286 (2020).

42. S. L. Freeman, H. Kwon, N. Portolano, G. Parkin, U. Venkatraman Girija, J. Basran, A. J. Fielding, L. Fairall, D. A. Svistunenko, P. C. E. Moody, J. W. R. Schwabe, C. P. Kyriacou, E. L. Raven, Heme binding to human CLOCK affects interactions with the E-box. *Proc. Natl. Acad. Sci. U.S.A.* **116**, 19911–19916 (2019).

43. E. M. Dioum, J. Rutter, J. R. Tuckerman, G. Gonzalez, M. A. Gilles-Gonzalez, S. L. McKnight, NPAS2: A gas-responsive transcription factor. *Science* **298**, 2385–2387 (2002).

44. M. V. Airola, J. Du, J. H. Dawson, B. R. Crane, Heme binding to the Mammalian circadian clock protein period 2 is nonspecific. *Biochemistry* **49**, 4327–4338 (2010).

45. U. Krauss, B. Q. Minh, A. Losi, W. Gärtner, T. Eggert, A. von Haeseler, K. E. Jaeger, Distribution and phylogeny of light-oxygen-voltage-blue-light-signaling proteins in the three kingdoms of life. *J. Bacteriol.* **191**, 7234–7242 (2009).

46. Z. J. Wang, S. M. Soohoo, P. B. Tiwari, G. Piszczek, T. I. Brelidze, Chlorpromazine binding to the PAS domains uncovers the effect of ligand modulation on EAG channel activity. *J. Biol. Chem.* **295**, 4114–4123 (2020).

47. J. Gruszczyk, L. Grandvuillemin, J. Lai-Kee-Him, M. Paloni, C. G. Savva, P. Germain, M. Grimaldi, A. Boulahtouf, H. S. Kwong, J. Bous, A. Ancelin, C. Bechara, A. Barducci, P. Balaguer, W. Bourguet, Cryo-EM structure of the agonist-bound Hsp90-XAP2-AHR cytosolic complex. *Nat. Commun.* **13**, 7010 (2022).

48. X. Ren, X. Diao, J. Zhuang, D. Wu, Structural basis for the allosteric inhibition of hypoxia-inducible factor 2 by belzutifan. *Mol. Pharmacol.* **102**, 240–247 (2022).

49. C. A. Amezcua, S. M. Harper, J. Rutter, K. H. Gardner, Structure and interactions of PAS kinase N-terminal PAS domain: Model for intramolecular kinase regulation. *Structure* **10**, 1349–1361 (2002).

50. J. P. McCutcheon, N. A. Moran, Extreme genome reduction in symbiotic bacteria. *Nat. Rev. Microbiol.* **10**, 13–26 (2011).

51. L. E. Ulrich, E. V. Koonin, I. B. Zhulin, One-component systems dominate signal transduction in prokaryotes. *Trends Microbiol.* **13**, 52–56 (2005).

52. J. Xing, V. M. Gumerov, I. B. Zhulin, Photoactive yellow protein represents a distinct, evolutionarily novel family of PAS domains. *J. Bacteriol.* **204**, e0030022 (2022).

53. M. Müllner, O. Hammel, B. Mienert, S. Schlag, E. Bill, G. Unden, A PAS domain with an oxygen labile $[4Fe\text{-}_4S]^{2+}$ cluster in the oxygen sensor kinase NreB of *Staphylococcus carnosus*. *Biochemistry* **47**, 13921–13932 (2008).

54. E. C. Stuffle, M. S. Johnson, K. J. Watts, PAS domains in bacterial signal transduction. *Curr. Opin. Microbiol.* **61**, 8–15 (2021).

55. Y. Liu, K. S. Makarova, W. C. Huang, Y. I. Wolf, A. N. Nikolskaya, X. Zhang, M. Cai, C. J. Zhang, W. Xu, Z. Luo, L. Cheng, E. V. Koonin, M. Li, Expanded diversity of Asgard archaea and their relationships with eukaryotes. *Nature* **593**, 553–557 (2021).

56. K. Wuichet, B. J. Cantwell, I. B. Zhulin, Evolution and phyletic distribution of two-component signal transduction systems. *Curr. Opin. Microbiol.* **13**, 219–225 (2010).

57. K. K. Koretke, A. N. Lupas, P. V. Warren, M. Rosenberg, J. R. Brown, Evolution of two-component signal transduction. *Mol. Biol. Evol.* **17**, 1956–1970 (2000).

58. V. Asher, H. Sowter, R. Shaw, A. Bali, R. Khan, Eag and HERG potassium channels as novel therapeutic targets in cancer. *World J. Surg. Oncol.* **8**, 113 (2010).

59. K. M. Brown, J. P. Day, E. Huston, B. Zimmermann, K. Hampel, F. Christian, D. Romano, S. Terhzaz, L. C. Lee, M. J. Willis, D. B. Morton, J. A. Beavo, M. Shimizu-Albergine, S. A. Davies, W. Kolch, M. D. Houslay, G. S. Baillie, Phosphodiesterase-8A binds to and regulates Raf-1 kinase. *Proc. Natl. Acad. Sci. U.S.A.* **110**, E1533–E1542 (2013).

60. J. Pinto, Y. P. Huang, R. S. Rivlin, Inhibition of riboflavin metabolism in rat tissues by chlorpromazine, imipramine, and amitriptyline. *J. Clin. Invest.* **67**, 1500–1506 (1981).

61. Z. Chen, A. Mondal, D. L. Minor Jr., Structural basis for $Ca_V\alpha_2\delta$:gabapentin binding. *Nat. Struct. Mol. Biol.*, **30**, 735–739 (2023).

62. K. M. Page, V. M. Gumerov, S. Dahimene, I. B. Zhulin, A. C. Dolphin, The importance of cache domains in $\alpha_2\delta$ proteins and the basis for their gabapentinoid selectivity. *Channels* **17**, 2167563 (2023).

63. S. Federhen, The NCBI Taxonomy database. *Nucleic Acids Res.* **40**, D136–D143 (2012).

64. D. H. Parks, M. Chuvochina, D. W. Waite, C. Rinke, A. Skarshewski, P. A. Chaumeil, P. Hugenholtz, A standardized bacterial taxonomy based on genome phylogeny substantially revises the tree of life. *Nat. Biotechnol.* **36**, 996–1004 (2018).

65. C. UniProt, UniProt: The universal protein knowledgebase in 2023. *Nucleic Acids Res.* **51**, D523–D531 (2023).

66. L. Fu, B. Niu, Z. Zhu, S. Wu, W. Li, CD-HIT: Accelerated for clustering the next-generation sequencing data. *Bioinformatics* **28**, 3150–3152 (2012).

67. A. Azad, G. A. Pavlopoulos, C. A. Ouzounis, N. C. Kyrpides, A. Buluc, HipMCL: A high-performance parallel implementation of the Markov clustering algorithm for large-scale networks. *Nucleic Acids Res.* **46**, e33 (2018).

68. K. Katoh, J. Rozewicki, K. D. Yamada, MAFFT online service: Multiple sequence alignment, interactive sequence choice and visualization. *Brief. Bioinform.* **20**, 1160–1166 (2019).

69. G. E. Crooks, G. Hon, J. M. Chandonia, S. E. Brenner, WebLogo: A sequence logo generator. *Genome Res.* **14**, 1188–1190 (2004).

70. T. J. Wheeler, J. Clements, R. D. Finn, Skylign: A tool for creating informative, interactive logos representing sequence alignments and profile hidden Markov models. *BMC Bioinformatics* **15**, 7 (2014).

71. V. M. Gumerov, I. B. Zhulin, TREND: A platform for exploring protein function in prokaryotes based on phylogenetic, domain architecture and gene neighborhood analyses. *Nucleic Acids Res.* **48**, W72–W76 (2020).

72. V. M. Gumerov, I. B. Zhulin, Correction to 'TREND: A platform for exploring protein function in prokaryotes based on phylogenetic, domain architecture and gene neighborhood analyses'. *Nucleic Acids Res.* **50**, 1795 (2022).

73. A. S. Konagurthu, J. C. Whisstock, P. J. Stuckey, A. M. Lesk, MUSTANG: A multiple structural alignment algorithm. *Proteins* **64**, 559–574 (2006).

74. B. Q. Minh, H. A. Schmidt, O. Chernomor, D. Schrempf, M. D. Woodhams, A. von Haeseler, R. Lanfear, IQ-TREE 2: New models and efficient methods for phylogenetic inference in the genomic Era. *Mol. Biol. Evol.* **37**, 1530–1534 (2020).

75. D. Darriba, G. L. Taboada, R. Doallo, D. Posada, ProtTest 3: Fast selection of best-fit models of protein evolution. *Bioinformatics* **27**, 1164–1165 (2011).

76. S. Mirarab, N. Nguyen, T. Warnow, SEPP: SATe-enabled phylogenetic placement. *Pac. Symp. Biocomput.*, 247–258 (2012).

77. K. Tunyasuvunakool, J. Adler, Z. Wu, T. Green, M. Zielinski, A. Žídek, A. Bridgland, A. Cowie, C. Meyer, A. Laydon, S. Velankar, G. J. Kleywegt, A. Bateman, R. Evans, A. Pritzel, M. Figurnov, O. Ronneberger, R. Bates, S. A. A. Kohl, A. Potapenko, A. J. Ballard, B. Romera-Paredes, S. Nikolov, R. Jain, E. Clancy, D. Reiman, S. Petersen, A. W. Senior, K. Kavukcuoglu, E. Birney, P. Kohli, J. Jumper, D. Hassabis, Highly accurate protein structure prediction for the human proteome. *Nature* **596**, 590–596 (2021).

78. Y. Zhang, J. Skolnick, TM-align: A protein structure alignment algorithm based on the TM-score. *Nucleic Acids Res.* **33**, 2302–2309 (2005).

79. K. J. Watts, B. L. Taylor, M. S. Johnson, PAS/poly-HAMP signalling in Aer-2, a soluble haem-based sensor. *Mol. Microbiol.* **79**, 686–699 (2011).

80. R. L. Kerby, H. Youn, G. P. Roberts, RcoM: A new single-component transcriptional regulator of CO metabolism in bacteria. *J. Bacteriol.* **190**, 3336–3343 (2008).

81. A. L. Chang, J. R. Tuckerman, G. Gonzalez, R. Mayer, H. Weinhouse, G. Volman, D. Amikam, M. Benziman, M. A. Gilles-Gonzalez, Phosphodiesterase A1, a regulator of cellulose synthesis in Acetobacter xylinum, is a heme-based sensor. *Biochemistry* **40**, 3420–3426 (2001).

82. F. Rao, Q. Ji, I. Soehano, Z. X. Liang, Unusual heme-binding PAS domain from YybT family proteins. *J. Bacteriol.* **193**, 1543–1551 (2011).

83. C. Carrica Mdel, I. Fernandez, M. A. Marti, G. Paris, F. A. Goldbaum, The NtrY/X two-component system of *Brucella* spp. acts as a redox sensor and regulates the expression of nitrogen respiration enzymes. *Mol. Microbiol.* **85**, 39–50 (2012).

84. J. Sarkar, D. P. Miller, L. D. Oliver Jr., R. T. Marconi, The treponema denticola PAS domain-containing histidine kinase Hpk2 is a heme binding sensor of oxygen levels. *J. Bacteriol.* **200**, e00116-18 (2018).

85. N. Singh, A. Kumar, Virulence factor SenX3 is the oxygen-controlled replication switch of Mycobacterium tuberculosis. *Antioxid. Redox Signal.* **22**, 603–613 (2015).

86. L. Yin, V. Dragnea, C. E. Bauer, PpsR, a regulator of heme and bacteriochlorophyll biosynthesis, is a heme-sensing protein. *J. Biol. Chem.* **287**, 13850–13858 (2012).

87. O. E. Petrova, K. Sauer, PAS domain residues and prosthetic group involved in BdlA-dependent dispersion response by Pseudomonas aeruginosa biofilms. *J. Bacteriol.* **194**, 5817–5828 (2012).

88. N. Jiang, W. Liu, Y. Li, H. Wu, Z. Zhang, G. Alexandre, C. Elmerich, Z. Xie, A chemotaxis receptor modulates nodulation during the azorhizobium caulinodans-sesbania rostrata symbiosis. *Appl. Environ. Microbiol.* **82**, 3174–3184 (2016).

89. K. Kitanishi, J. Igarashi, K. Hayasaka, N. Hikage, I. Saiful, S. Yamauchi, T. Uchida, K. Ishimori, T. Shimizu, Heme-binding characteristics of the isolated PAS-A domain of mouse Per2, a transcriptional regulatory factor associated with circadian rhythms. *Biochemistry* **47**, 6157–6168 (2008).

90. K. Hayasaka, K. Kitanishi, J. Igarashi, T. Shimizu, Heme-binding characteristics of the isolated PAS-B domain of mouse Per2, a transcriptional regulatory factor associated with circadian rhythms. *Biochim. Biophys. Acta* **1814**, 326–333 (2011).

91. A. Rebbapragada, M. S. Johnson, G. P. Harding, A. J. Zuccarelli, H. M. Fletcher, I. B. Zhulin, B. L. Taylor, The Aer protein and the serine chemoreceptor Tsr independently sense intracellular energy levels and transduce oxygen, redox, and energy signals for Escherichia coli behavior. *Proc. Natl. Acad. Sci. U.S.A.* **94**, 10541–10546 (1997).

92. K. T. Elliott, V. J. Dirita, Characterization of CetA and CetB, a bipartite energy taxis system in Campylobacter jejuni. *Mol. Microbiol.* **69**, 1091–1103 (2008).

93. A. T. Vaidya, C. H. Chen, J. C. Dunlap, J. J. Loros, B. R. Crane, Structure of a light-activated LOV protein dimer that regulates transcription. *Sci. Signal.* **4**, ra50 (2011).

94. Q. He, P. Cheng, Y. Yang, L. Wang, K. H. Gardner, Y. Liu, White collar-1, a DNA binding transcription factor and a light sensor. *Science* **297**, 840–843 (2002).

95. A. Losi, E. Polverini, B. Quest, W. Gartner, First evidence for phototropin-related blue-light receptors in prokaryotes. *Biophys. J.* **82**, 2627–2634 (2002).

96. M. Nakasako, K. Zikihara, D. Matsuoka, H. Katsura, S. Tokutomi, Structural basis of the LOV1 dimerization of Arabidopsis phototropins 1 and 2. *J. Mol. Biol.* **381**, 718–733 (2008).

97. S. Crosson, K. Moffat, Structure of a flavin-binding plant photoreceptor domain: Insights into light-mediated signal transduction. *Proc. Natl. Acad. Sci. U.S.A.* **98**, 2995–3000 (2001).

98. A. Hagman, L. X. Shi, E. Rintamaki, B. Andersson, W. P. Schroder, The nuclear-encoded PsbW protein subunit of photosystem II undergoes light-induced proteolysis. *Biochemistry* **36**, 12666–12671 (1997).

99. K. Röllen, J. Granzin, V. Panwalkar, V. Arinkin, R. Rani, R. Hartmann, U. Krauss, K.-E. Jaeger, D. Willbold, R. Batra-Safferling, Signaling states of a short blue-light photoreceptor protein PpSB1-LOV revealed from crystal structures and solution NMR spectroscopy. *J. Mol. Biol.* **428**, 3721–3736 (2016).

100. S. Endres, J. Granzin, F. Circolone, A. Stadler, U. Krauss, T. Drepper, V. Svensson, E. Knieps-Grünhagen, A. Wirtz, A. Cousin, P. Tielen, D. Willbold, K. E. Jaeger, R. Batra-Safferling, Structure and function of a short LOV protein from the marine phototrophic bacterium Dinoroseobacter shibae. *BMC Microbiol.* **15**, 30 (2015).

101. E. B. Purcell, D. Siegal-Gaskins, D. C. Rawling, A. Fiebig, S. Crosson, A photosensory two-component system regulates bacterial cell attachment. *Proc. Natl. Acad. Sci. U.S.A.* **104**, 18241–18246 (2007).

102. T. E. Swartz, T. S. Tseng, M. A. Frederickson, Ǵ. Paris, D. J. Comerci, G. Rajashekara, J. G. Kim, M. B. Mudgett, G. A. Splitter, R. A. Ugalde, F. A. Goldbaum, W. R. Briggs, R. A. Bogomolni, Blue-light-activated histidine kinases: Two-component sensors in bacteria. *Science* **317**, 1090–1093 (2007).

103. A. I. Nash, R. McNulty, M. E. Shillito, T. E. Swartz, R. A. Bogomolni, H. Luecke, K. H. Gardner, Structural basis of photosensitivity in a bacterial light-oxygen-voltage/helix-turn-helix (LOV-HTH) DNA-binding protein. *Proc. Natl. Acad. Sci. U.S.A.* **108**, 9449–9454 (2011).

104. V. V. Nazarenko, A. Remeeva, A. Yudenko, K. Kovalev, A. Dubenko, I. M. Goncharov, P. Kuzmichev, A. V. Rogachev, P. Buslaev, V. Borshchevskiy, A. Mishin, G. V. Dhoke, U. Schwaneberg, M. D. Davari, K. E. Jaeger, U. Krauss, V. Gordeliy, I. Gushchin, A thermostable flavin-based fluorescent protein from Chloroflexus aggregans: A framework for ultra-high resolution structural studies. *Photochem. Photobiol. Sci.* **18**, 1793–1805 (2019).

105. U. Heintz, I. Schlichting, Blue light-induced LOV domain dimerization enhances the affinity of Aureochrome 1a for its target DNA sequence. *eLife* **5**, e11860 (2016).

106. A. M. Weber, J. Kaiser, T. Ziegler, S. Pilsl, C. Renzl, L. Sixt, G. Pietruschka, S. Moniot, A. Kakoti, M. Juraschitz, S. Schrottke, L. Lledo Bryant, C. Steegborn, R. Bittl, G. Mayer, A. Möglich, A blue light receptor that mediates RNA binding and translational regulation. *Nat. Chem. Biol.* **15**, 1085–1092 (2019).

107. J. Lokhandwala, H. C. Hopkins, A. Rodriguez-Iglesias, C. Dattenböck, M. Schmoll, B. D. Zoltowski, Structural biochemistry of a fungal LOV domain photoreceptor reveals an evolutionarily conserved pathway integrating light and oxidative stress. *Structure* **23**, 116–125 (2015).

108. Z. Jiang, L. R. Swem, B. G. Rushing, S. Devanathan, G. Tollin, C. E. Bauer, Bacterial photoreceptor with similarity to photoactive yellow protein and plant phytochromes. *Science* **285**, 406–409 (1999).

109. J. A. Kyndt, J. C. Fitch, T. E. Meyer, M. A. Cusanovich, Thermochromatium tepidum photoactive yellow protein/bacteriophytochrome/diguanylate cyclase: Characterization of the PYP domain. *Biochemistry* **44**, 4755–4764 (2005).

110. M. E. Curran, I. Splawski, K. W. Timothy, G. M. Vincen, E. D. Green, M. T. Keating, A molecular basis for cardiac arrhythmia: HERG mutations cause long QT syndrome. *Cell* **80**, 795–803 (1995).

111. R. Brugada, K. Hong, R. Dumaine, J. Cordeiro, F. Gaita, M. Borggrefe, T. M. Menendez, J. Brugada, G. D. Pollevick, C. Wolpert, E. Burashnikov, K. Matsuo, Y. Sheng Wu, A. Guerchicoff, F. Bianchi, C. Giustetto, R. Schimpf, P. Brugada, C. Antzelevitch, Sudden death associated with short-QT syndrome linked to mutations in HERG. *Circulation* **109**, 30–35 (2004).

112. W. Shi, R. S. Wymore, H. S. Wang, Z. Pan, I. S. Cohen, D. McKinnon, J. E. Dixon, Identification of two nervous system-specific members of the erg potassium channel gene family. *J. Neurosci.* **17**, 9423–9432 (1997).

113. L. A. Pardo, W. Stuhmer, The roles of K(+) channels in cancer. *Nat. Rev. Cancer* **14**, 39–48 (2014).

114. C. Simons, L. D. Rash, J. Crawford, L. Ma, B. Cristofori-Armstrong, D. Miller, K. Ru, G. J. Baillie, Y. Alanay, A. Jacquinet, F. G. Debray, A. Verloes, J. Shen, G. Yesil, S. Guler, A. Yuksel, J. G. Cleary, S. M. Grimmond, J. McGaughran, G. F. King, M. T. Gabbett, R. J. Taft, Mutations in the voltage-gated potassium channel gene KCNH1 cause Temple-Baraitser syndrome and epilepsy. *Nat. Genet.* **47**, 73–77 (2015).

115. F. Kortüm, V. Caputo, C. K. Bauer, L. Stella, A. Ciolfi, M. Alawi, G. Bocchinfuso, E. Flex, S. Paolacci, M. L. Dentici, P. Grammatico, G. C. Korenke, V. Leuzzi, D. Mowat, L. D. V. Nair, T. T. M. Nguyen, P. Thierry, S. M. White, B. Dallapiccola, A. Pizzuti, P. M. Campeau, M. Tartaglia, K. Kutsche, Mutations in KCNH1 and ATP6V1B2 cause Zimmermann-Laband syndrome. *Nat. Genet.* **47**, 661–667 (2015).

116. X. Huang, Y. He, A. M. Dubuc, R. Hashizume, W. Zhang, J. Reimand, H. Yang, T. A. Wang, S. J. Stehbens, S. Younger, S. Barshow, S. Zhu, M. K. Cooper, J. Peacock, V. Ramaswamy, L. Garzia, X. Wu, M. Remke, C. M. Forester, C. C. Kim, W. A. Weiss, C. D. James, M. A. Shuman, G. D. Bader, S. Mueller, M. D. Taylor, Y. N. Jan, L. Y. Jan, EAG2 potassium channel with evolutionarily conserved function as a brain tumor target. *Nat. Neurosci.* **18**, 1236–1246 (2015).

117. B. Engeland, A. Neu, J. Ludwig, J. Roeper, O. Pongs, Cloning and functional expression of rat *ether-à-go-go*-like K^+ channel genes. *J. Physiol.* **513** (Pt 3), 647–654 (1998).

118. A. Zou, Z. Lin, M. Humble, C. D. Creech, P. K. Wagoner, D. Krafte, T. J. Jegla, A. D. Wickenden, Distribution and functional properties of human KCNH8 (Elk1) potassium channels. *Am. J. Physiol. Cell Physiol.* **285**, C1356–C1366 (2003).

119. F. Chimienti, L. Cavarec, L. Vincent, N. Salvetat, V. Arango, M. D. Underwood, J. J. Mann, J. F. Pujol, D. Weissmann, Brain region-specific alterations of RNA editing in PDE8A mRNA in suicide decedents. *Transl. Psychiatry* **9**, 91 (2019).

120. A. Horvath, V. Mericq, C. A. Stratakis, Mutation in PDE8B, a cyclic AMP-specific phosphodiesterase in adrenal hyperplasia. *N. Engl. J. Med.* **358**, 750–752 (2008).

121. Y. Li, S. Innocentin, D. R. Withers, N. A. Roberts, A. R. Gallagher, E. F. Grigorieva, C. Wilhelm, M. Veldhoen, Exogenous stimuli maintain intraepithelial lymphocytes via aryl hydrocarbon receptor activation. *Cell* **147**, 629–640 (2011).

122. V. Rothhammer, F. J. Quintana, The aryl hydrocarbon receptor: An environmental sensor integrating immune responses in health and disease. *Nat. Rev. Immunol.* **19**, 184–197 (2019).

123. E. Zudaire, N. Cuesta, V. Murty, K. Woodson, L. Adams, N. Gonzalez, A. Martínez, G. Narayan, I. Kirsch, W. Franklin, F. Hirsch, M. Birrer, F. Cuttitta, The aryl hydrocarbon receptor repressor is a putative tumor suppressor gene in multiple human cancers. *J. Clin. Investig.* **118**, 640–650 (2008).

124. G. L. Semenza, Hypoxia-inducible factor 1 and cardiovascular disease. *Annu. Rev. Physiol.* **76**, 39–56 (2014).

125. W. Chen, H. Hill, A. Christie, M. S. Kim, E. Holloman, A. Pavia-Jimenez, F. Homayoun, Y. Ma, N. Patel, P. Yell, G. Hao, Q. Yousuf, A. Joyce, I. Pedrosa, H. Geiger, H. Zhang, J. Chang, K. H. Gardner, R. K. Bruick, C. Reeves, T. H. Hwang, K. Courtney, E. Frenkel, X. Sun, N. Zojwalla, T. Wong, J. P. Rizzi, E. M. Wallace, J. A. Josey, Y. Xie, X. J. Xie, P. Kapur, R. M. McKay, J. Brugarolas, Targeting renal cell carcinoma with a HIF-2 antagonist. *Nature* **539**, 112–117 (2016).

126. M. J. Percy, P. W. Furlow, G. S. Lucas, X. Li, T. R. J. Lappin, M. F. McMullin, F. S. Lee, A gain-of-function mutation in the HIF2A gene in familial erythrocytosis. *N. Engl. J. Med.* **358**, 162–168 (2008).

127. X. Zhou, X. Guo, M. Chen, C. Xie, J. Jiang, HIF-3α promotes metastatic phenotypes in pancreatic cancer by transcriptional regulation of the RhoC-ROCK1 signaling pathway. *Mol. Cancer Res.* **16**, 124–134 (2018).

128. Y. Makino, R. Cao, K. Svensson, G. Bertilsson, M. Asman, H. Tanaka, Y. Cao, A. Berkenstam, L. Poellinger, Inhibitory PAS domain protein is a negative regulator of hypoxia-inducible gene expression. *Nature* **414**, 550–554 (2001).

129. S. Torii, S. Kasai, A. Suzuki, Y. Todoroki, K. Yokozawa, K. I. Yasumoto, N. Seike, H. Kiyonari, Y. Mukumoto, A. Kakita, K. Sogawa, Involvement of inhibitory PAS domain protein in neuronal cell death in Parkinson's disease. *Cell Death Discov.* **1**, 15015 (2015).

130. J. L. Michaud, F. Boucher, A. Melnyk, F. Gauthier, E. Goshu, E. Lévy, G. A. Mitchell, J. Himms-Hagen, C. M. Fan, Sim1 haploinsufficiency causes hyperphagia, obesity and reduction of the paraventricular nucleus of the hypothalamus. *Hum. Mol. Genet.* **10**, 1465–1473 (2001).

131. H. Chen, R. Chrast, C. Rossier, A. Gos, S. E. Antonarakis, J. Kudoh, A. Yamaki, N. Shindoh, H. Maeda, S. Minoshima, N. Shimizu, Single-minded and Down syndrome? *Nat. Genet.* **10**, 9–10 (1995).

132. N. Dahmane, G. Charron, C. Lopes, M. L. Yaspo, C. Maunoury, L. Decorte, P. M. Sinet, B. Bloch, J. M. Delabar, Down syndrome-critical region contains a gene homologous to Drosophila sim expressed during rat and human central nervous system development. *Proc. Natl. Acad. Sci. U.S.A.* **92**, 9191–9195 (1995).

133. M. P. DeYoung, M. Tress, R. Narayanan, Identification of Down's syndrome critical locus gene SIM2-s as a drug therapy target for solid tumors. *Proc. Natl. Acad. Sci. U.S.A.* **100**, 4760–4765 (2003).

134. Y. D. Zhou, M. Barnard, H. Tian, X. Li, H. Z. Ring, U. Francke, J. Shelton, J. Richardson, D. W. Russell, S. L. McKnight, Molecular characterization of two mammalian bHLH-PAS domain proteins selectively expressed in the central nervous system. *Proc. Natl. Acad. Sci. U.S.A.* **94**, 713–718 (1997).

135. J. J. Michaelson, M. K. Shin, J. Y. Koh, L. Brueggeman, A. Zhang, A. Katzman, L. McDaniel, M. Fang, M. Pufall, A. A. Pieper, Neuronal PAS domain proteins 1 and 3 are master regulators of neuropsychiatric risk genes. *Biol. Psychiatry* **82**, 213–223 (2017).

136. L. Sha, L. MacIntyre, J. A. Machell, M. P. Kelly, D. J. Porteous, N. J. Brandon, W. J. Muir, D. H. Blackwood, D. G. Watson, S. J. Clapcote, B. S. Pickard, Transcriptional regulation of neurodevelopmental and metabolic pathways by NPAS3. *Mol. Psychiatry* **17**, 267–279 (2012).

137. J. Wong, C. E. Duncan, N. J. Beveridge, M. J. Webster, M. J. Cairns, C. Shannon Weickert, Expression of NPAS3 in the human cortex and evidence of its posttranscriptional regulation by miR-17 during development, with implications for schizophrenia. *Schizophr. Bull.* **39**, 396–406 (2013).

138. R. Shepard, K. Heslin, P. Hagerdorn, L. Coutellier, Downregulation of Npas4 in parvalbumin interneurons and cognitive deficits after neonatal NMDA receptor blockade: Relevance for schizophrenia. *Transl. Psychiatry* **9**, 99 (2019).

139. P. V. Sabatini, T. Speckmann, C. Nian, M. M. Glavas, C. K. Wong, J. S. Yoon, T. Kin, A. M. J. Shapiro, W. T. Gibson, C. B. Verchere, F. C. Lynn, Neuronal PAS domain protein 4 suppression of oxygen sensing optimizes metabolism during excitation of neuroendocrine cells. *Cell Rep.* **22**, 163–174 (2018).

140. E. A. Pollina, D. T. Gilliam, A. T. Landau, C. Lin, N. Pajarillo, C. P. Davis, D. A. Harmin, E. L. Yap, I. R. Vogel, E. C. Griffith, M. A. Nagy, E. Ling, E. E. Duffy, B. L. Sabatini, C. J. Weitz, M. E. Greenberg, A NPAS4-NuA4 complex couples synaptic activity to DNA repair. *Nature,* **614**, 732–741 (2023).

141. N. Gekakis, D. Staknis, H. B. Nguyen, F. C. Davis, L. D. Wilsbacher, D. P. King, J. S. Takahashi, C. J. Weitz, Role of the CLOCK protein in the mammalian circadian mechanism. *Science* **280**, 1564–1569 (1998).

142. M. Reick, J. A. Garcia, C. Dudley, S. L. McKnight, NPAS2: An analog of clock operative in the mammalian forebrain. *Science* **293**, 506–509 (2001).

143. A. K. Michael, S. L. Harvey, P. J. Sammons, A. P. Anderson, H. M. Kopalle, A. H. Banham, C. L. Partch, Cancer/Testis Antigen PASD1 silences the circadian clock. *Mol. Cell* **58**, 743–754 (2015).

144. S. S. Sahota, C. M. Goonewardena, C. D. O. Cooper, A. P. Liggins, K. Ait-Tahar, N. Zojer, F. K. Stevenson, A. H. Banham, K. Pulford, PASD1 is a potential multiple myeloma-associated antigen. *Blood* **108**, 3953–3955 (2006).

145. C. D. O. Cooper, A. P. Liggins, K. Ait-Tahar, G. Roncador, A. H. Banham, K. Pulford, PASD1, a DLBCL-associated cancer testis antigen and candidate for lymphoma immunotherapy. *Leukemia* **20**, 2172–2174 (2006).

146. D. C. Bersten, A. E. Sullivan, D. J. Peet, M. L. Whitelaw, bHLH-PAS proteins in cancer. *Nat. Rev. Cancer* **13**, 827–841 (2013).

147. M. K. Bunger, L. D. Wilsbacher, S. M. Moran, C. Clendenin, L. A. Radcliffe, J. B. Hogenesch, M. C. Simon, J. S. Takahashi, C. A. Bradfield, Mop3 is an essential component of the master circadian pacemaker in mammals. *Cell* **103**, 1009–1017 (2000).

148. S. Shi, A. Hida, O. P. McGuinness, D. H. Wasserman, S. Yamazaki, C. H. Johnson, Circadian clock gene Bmal1 is not essential; functional replacement with its paralog, Bmal2. *Curr. Biol.* **20**, 316–321 (2010).

149. S. A. Onate, S. Y. Tsai, M. J. Tsai, B. W. O'Malley, Sequence and characterization of a coactivator for the steroid hormone receptor superfamily. *Science* **270**, 1354–1357 (1995).

150. S. L. Anzick, J. Kononen, R. L. Walker, D. O. Azorsa, M. M. Tanner, X. Y. Guan, G. Sauter, O. P. Kallioniemi, J. M. Trent, P. S. Meltzer, AIB1, a steroid receptor coactivator amplified in breast and ovarian cancer. *Science* **277**, 965–968 (1997).

151. B. W. O'Malley, SRC-2 Coactivator: A role in human metabolic evolution and disease. *Mol. Med.* **26**, 45 (2020).

152. A. Gupta, M. M. Hossain, N. Miller, M. Kerin, G. Callagy, S. Gupta, NCOA3 coactivator is a transcriptional target of XBP1 and regulates PERK–eIF2α–ATF4 signalling in breast cancer. *Oncogene* **35**, 5860–5871 (2016).

153. R. Salazar-Silva, V. L. G. Dantas, L. U. Alves, A. C. Batissoco, J. Oiticica, E. A. Lawrence, A. Kawafi, Y. Yang, F. S. Nicastro, B. C. Novaes, C. Hammond, E. Kague, R. C. Mingroni-Netto, NCOA3 identified as a new candidate to explain autosomal dominant progressive hearing loss (vol 29, pg 3691, 2020). *Hum. Mol. Genet.* **31**, 156 (2022).

154. J. L. Kwapis, Y. Alaghband, E. A. Kramár, A. J. López, A. Vogel Ciernia, A. O. White, G. Shu, D. Rhee, C. M. Michael, E. Montellier, Y. Liu, C. N. Magnan, S. Chen, P. Sassone-Corsi, P. Baldi, D. P. Matheos, M. A. Wood, Epigenetic regulation of the circadian gene Per1 contributes to age-related changes in hippocampal memory. *Nat. Commun.* **9**, 3323 (2018).

155. A. Zietara, D. R. Spires, A. Juffre, H. M. Costello, G. R. Crislip, L. G. Douma, V. Levchenko, L. V. Dissanayake, C. A. Klemens, O. Nikolaienko, A. M. Geurts, M. L. Gumz, A. Staruschenko, Knockout of the circadian clock protein PER1 (Period1) exacerbates hypertension and increases kidney injury in Dahl salt-sensitive rats. *Hypertension* **79**, 2519–2529 (2022).

156. H. Viswambharan, J̃. M. Carvas, V. Antic, A. Marecic, C. Jud, C. E. Zaugg, X. F. Ming, J. P. Montani, U. Albrecht, Z. Yang, Mutation of the circadian clock gene Per2 alters vascular endothelial function. *Circulation* **115**, 2188–2195 (2007).

157. L. Zhang, A. Hirano, P. K. Hsu, C. R. Jones, N. Sakai, M. Okuro, T. McMahon, M. Yamazaki, Y. Xu, N. Saigoh, K. Saigoh, S. T. Lin, K. Kaasik, S. Nishino, L. J. Ptáček, Y. H. Fu, A PERIOD3 variant causes a circadian phenotype and is associated with a seasonal mood trait. *Proc. Natl. Acad. Sci. U.S.A.* **113**, E1536–E1544 (2016).

158. H. X. Hao, C. M. Cardon, W. Swiatek, R. C. Cooksey, T. L. Smith, J. Wilde, S. Boudina, E. D. Abel, D. A. McClain, J. Rutter, PAS kinase is required for normal cellular energy balance. *Proc. Natl. Acad. Sci. U.S.A.* **104**, 15466–15471 (2007).
